# The Australian National University Alzheimer's Disease Risk Index (ANU‐ADRI) score as a predictor for cognitive decline and potential surrogate outcome in the FINGER lifestyle randomized controlled trial

**DOI:** 10.1111/ene.16238

**Published:** 2024-02-07

**Authors:** Anette Hall, Mariagnese Barbera, Jenni Lehtisalo, Riitta Antikainen, Hamidul Huque, Tiina Laatikainen, Tiia Ngandu, Hilkka Soininen, Ruth Stephen, Timo Strandberg, Miia Kivipelto, Kaarin J. Anstey, Alina Solomon

**Affiliations:** ^1^ Department of Neurology, Institute of Clinical Medicine University of Eastern Finland Kuopio Finland; ^2^ Division of Clinical Geriatrics, Center for Alzheimer Research, Department of Neurobiology, Care Sciences and Society Karolinska Institutet Stockholm Sweden; ^3^ Ageing Epidemiology Research Unit, School of Public Health Imperial College London London UK; ^4^ Population Health Unit, Department of Public Health and Welfare Finnish Institute for Health and Welfare Helsinki Finland; ^5^ Center for Life Course Health Research/Geriatrics University of Oulu Oulu Finland; ^6^ Medical Research Center Oulu University Hospital Oulu Finland; ^7^ School of Psychology University of New South Wales, Sydney Sydney New South Wales Australia; ^8^ Neuroscience Research Australia Randwick New South Wales Australia; ^9^ Institute of Public Health and Clinical Nutrition University of Eastern Finland Kuopio Finland; ^10^ Neurocenter Finland, Department of Neurology Kuopio University Hospital Kuopio Finland; ^11^ University of Helsinki and Helsinki University Hospital Helsinki Finland; ^12^ Center for Life Course Health Research University of Oulu Oulu Finland; ^13^ Theme Inflammation and Aging Karolinska university hospital Stockholm Sweden

**Keywords:** Alzheimer's, clinical trials, cognition

## Abstract

**Background and purpose:**

The complex aetiology of Alzheimer's disease suggests prevention potential. Risk scores have potential as risk stratification tools and surrogate outcomes in multimodal interventions targeting specific at‐risk populations. The Australian National University Alzheimer's Disease Risk Index (ANU‐ADRI) was tested in relation to cognition and its suitability as a surrogate outcome in a multidomain lifestyle randomized controlled trial, in older adults at risk of dementia.

**Methods:**

In this post hoc analysis of the Finnish Intervention Study to Prevent Cognitive Impairment and Disability (FINGER), ANU‐ADRI was calculated at baseline, 12, and 24 months (*n* = 1174). The association between ANU‐ADRI and cognition (at baseline and over time), the intervention effect on changes in ANU‐ADRI, and the potential impact of baseline ANU‐ADRI on the intervention effect on changes in cognition were assessed using linear mixed models with maximum likelihood estimation.

**Results:**

A higher ANU‐ADRI was significantly related to worse cognition, at baseline (e.g., estimate for global cognition [95% confidence interval] was −0.028 [−0.032 to −0.025]) and over the 2‐year study (e.g., estimate for 2‐year changes in ANU‐ADRI and per‐year changes in global cognition [95% confidence interval] was −0.068 [−0.026 to −0.108]). No significant beneficial intervention effect was reported for ANU‐ADRI, and baseline ANU‐ADRI did not significantly affect the response to the intervention on changes in cognition.

**Conclusions:**

The ANU‐ADRI was effective for the risk prediction of cognitive decline. Risk scores may be crucial for the success of novel dementia prevention strategies, but their algorithm, the target population, and the intervention design should be carefully considered when choosing the appropriate tool for each context.

## INTRODUCTION

The complex aetiology of Alzheimer's disease (AD), featuring many interconnected modifiable risk factors and a slow onset [1], suggests prevention potential [2]. So far, most of the drug trials have not resulted in effective disease‐modifying therapies [3]. Although promising, preventive interventions targeting modifiable risk factors reported inconclusive results [4, 5, 6]. This could depend on the choice of target population, which could either have a risk profile not sufficiently matched to the intervention or be at an advanced disease stage that requires additional pharmacological therapy. Limitations in the intervention design, such as insufficient intensity and lack of tailoring to the specific at‐risk group, have been identified as crucial [7] and might have contributed to these unclear results. Precision approaches in specific at‐risk groups and/or addressing multiple risk factors simultaneously through multidomain interventions could have great potential but are still very rare in dementia prevention. When designing effective novel prevention programmes, the choice of the appropriate tools is instrumental to identify the populations who could benefit the most, plan interventions that are tailored to specific risk profiles, estimate the intervention's prevention potential in a shorter time frame than that of dementia onset, and monitor dementia risk trends and changes in discrete populations [7].

In this context, the potential of risk scores, that is, a weighted composite of risk factors reflecting individuals' likelihood of developing dementia, has been identified [8]. Risk scores could play a critical dual role: as risk stratification tools [9], to enable the selection of the most appropriate target population when used to assess eligibility, and as estimated measures of dementia risk, to capture the risk reduction potential of a certain intervention when used as surrogate outcome [10, 11, 12].

The Australian National University Alzheimer's Disease Risk Index (ANU‐ADRI) is a questionnaire‐based risk score for AD (http://anuadri.anu.edu.au). It was developed on the basis of a systematic review of high‐quality evidence from meta‐analyses or cohort studies of established risk or protective factors [13], and its predicting potential for AD, dementia and mild cognitive impairment has been extensively validated [14, 15]. ANU‐ADRI has been successfully used as a selection tool to identify at‐risk individuals as suitable trial participants, as well as a surrogate outcome in small randomized controlled trials (RCTs) testing multidomain preventive intervention in different populations and contexts [16, 17, 18, 19]. ANU‐ADRI is a secondary outcome in an ongoing primary prevention internet‐based trial [20] and has been proposed as an effective risk prediction and surrogate outcome tool in larger RCTs and public health programmes [8, 21].

The aim was to test ANU‐ADRI in relation to cognitive decline and its suitability as a surrogate outcome to capture beneficial effects of a multidomain lifestyle preventive intervention, in older adults at increased risk of dementia. Data from the Finnish Intervention Study to Prevent Cognitive Impairment and Disability (FINGER), the first large multidomain RCT showing the efficacy of a multidomain lifestyle intervention in preventing cognitive decline, were used. In particular, (1) the association between ANU‐ADRI and cognition during FINGER; (2) the effect of the FINGER intervention on changes in ANU‐ADRI; and (3) the potential modifying effect of baseline ANU‐ADRI on the response to FINGER intervention on cognition were investigated.

## METHODS

### Study sample and settings

The present study is a post hoc analysis of the FINGER trial, which has been described in detail previously [22, 23]. In brief, between 2009 and 2011, 1260 participants aged 60–77 years were recruited across Finland from earlier population‐based health surveys. Eligible participants had a Cardiovascular Risk Factors, Ageing and Dementia (CAIDE) score of at least 6 points, indicating the presence of modifiable risk factors, and cognitive performance at the mean level or slightly below the mean of the respective age group, based on the Consortium to Establish a Registry for Alzheimer's Disease cognitive screening test. Individuals with dementia or substantial cognitive impairment, based on the Mini‐Mental State Examination <20 or clinical judgement, conditions preventing safe engagement in the lifestyle intervention or individuals participating in another ongoing intervention study were excluded.

The primary outcome of the trial was changes in cognitive function, which were measured using an extended version of the neuropsychological test battery (NTB) (including 14 tests). Change in cognitive domains included in the NTB were included amongst the secondary outcomes.

At baseline, participants were randomized 1:1 to the intervention (*N* = 631) or control (*N* = 629) groups. The intervention group received an intensive lifestyle programme structured around four main components (healthy diet, physical exercise, cognitive training, and management of vascular and metabolic risk factors), with social activities included in the form of group meetings as part of the delivery of the main intervention's components. The control group received regular health advice. The intervention duration was 2 years.

The present study includes all FINGER participants, for whom full data were available for the calculation of the ANU‐ADRI risk score (*N* = 1174) at baseline.

The study was conducted according to the guidelines of the Declaration of Helsinki. FINGER was approved by the Coordinating Ethics Committee of the Hospital District of Helsinki and Uusimaa (nr. 94/13/03/00/2009, 7 April 2009). Written informed consent was obtained from all participants at screening and baseline visit. FINGER is registered at ClinicalTrials.gov (no. NCT01041989).

### Data collection and outcome measures

#### Australian National University Alzheimer's Disease Risk Index

Development and composition of the ANU‐ADRI risk score have been described previously [13]. In brief, it includes 15 components: age, sex, education, body mass index (BMI), depression, diabetes, cholesterol, smoking, traumatic brain injury, physical activity, cognitive activity, social engagement, alcohol intake, dietary fish intake, and pesticide exposure. For each factor, estimates from existing publications were used to allocate specific points, which were weighted relative to the factor's effect size, based on the self‐reported data. The overall score is generated by the sum of all factors' points. Positive points are allocated to each risk factor (e.g., smoking status), whereas negative points are allocated to protective factors (e.g., physical activity), so that a higher ANU‐ADRI risk score indicates a higher risk of AD.

In the present study, the ANU‐ADRI risk score was calculated based on self‐reported or measured data, collected as described previously [22], from 12 factors. Information on age and sex were derived from the Finnish national population register (https://dvv.fi/en). In the ANU‐ADRI algorithm, BMI and blood cholesterol are included only for individuals aged 60 years or younger and therefore were omitted from this analysis. Information on pesticide exposure was not available. In FINGER, social engagement and cognitive activity were self‐reported using different questions than those originally used in the ANU‐ADRI questionnaire (Supplementary Methods). In order to capture potential differences in the role of risk versus protective factors, sub‐scores including either only risk or only protective factors were also considered. Furthermore, the combination of non‐modifiable age and sex has a substantial weight in the overall ANU‐ADRI risk score, due to being used as a categorical, rather than linear, function with considerable sub‐scores difference amongst discrete categories, and could mask potential score changes linked to the modification of other risk/protective factors. Therefore, a version of the score without age and sex was also tested when changes in the ANU‐ADRI risk score over time were considered. Details of the specific algorithm used in this study to calculate the ANU‐ADRI risk score are described in Table S1.

#### Cognition

The FINGER cognitive assessment has been described in detail previously [22]. In brief, the NTB (Table S2) was administered, during the 2‐year intervention, at baseline and 12 and 24 months by study psychologists who were blinded to the intervention allocation. Test results for each time point were calculated on a *z*‐scale standardized to the baseline mean and standard deviation, with higher scores indicating better performance. Zero‐skewness log‐transformation was applied to skewed NTB components. In the present study, both the NTB composite *z*‐score (primary outcome of the trial) and domain‐specific NTB *z*‐scores for memory, processing speed and executive function (secondary cognitive outcomes) were included. The NTB composite *z*‐score was calculated when at least eight of 14 test results were available and, respectively, three of six for memory, two of three for processing speed, and three of five for executive function.

### Statistical analysis

Baseline differences between the intervention and control groups in demographics, cognition, individual risk and protective factors, the ANU‐ADRI risk score and its sub‐scores were examined with the *t* test, rank sum and *χ* ^2^ test, as appropriate. Linear mixed models with maximum likelihood estimation were used to assess (1) the association between ANU‐ADRI and cognition, at baseline and over time, (2) the intervention effect on changes in ANU‐ADRI and its risk/protective sub‐scores, and (3) the potential impact of baseline ANU‐ADRI on the intervention effect on changes in cognition.

The association between ANU‐ADRI and cognition was assessed (a) cross‐sectionally at baseline, and over time testing the association between (b) baseline ANU‐ADRI and yearly changes in cognition and (c) dichotomous change in ANU‐ADRI and yearly changes in cognition. A dichotomous ANU‐ADRI change variable was generated and coded as ‘decreased’ if the change was <0 or ‘stable/increased’ if the change was ≥0, compared to baseline. As described above, the combination of age and sex was not included when changes in the ANU‐ADRI risk score were considered. The model included randomization group, time in years, baseline ANU‐ADRI risk score, and the interactions time × randomization group, time × baseline ANU‐ADRI risk score and time × dichotomous changes in ANU‐ADRI risk scores, adjusted for study site.

Mixed model repeated measures with maximum likelihood estimation were used to assess intervention effect on changes in ANU‐ADRI as full score, an adapted version without age and sex, as well as sub‐scores that included either its risk or protective components alone. Models included randomization group, time, group × time interaction and study site.

The modifying effect of baseline ANU‐ADRI risk score on the intervention effect on changes in cognition was assessed using a model including time; randomization group; baseline ANU‐ADRI; the ANU‐ADRI × time; the ANU‐ADRI × group; group × time; and time × group × ANU‐ADRI interactions; and study site. The modifying effect was described by the three‐way interaction term of time × group × ANU‐ADRI.

All analyses were done in Stata/SE 14 and 15 (StataCorp, TX, USA), and the level of statistical significance was *p* < 0.05 in two‐sided tests.

## RESULTS

### Baseline population description

Within the original FINGER cohort (*N* = 1259) [23], a total of 1174 participants were included in the present analysis (Table 1). At baseline, no statistical difference was identified between intervention and control arms, except for the ANU‐ADRI sub‐score including risk factors only (*p* = 0.04). The baseline ANU‐ADRI risk score of participants without available 2‐year ANU‐ADRI data (199 out of 1174; mean 8.6, SD 8.6) was higher than that of those who completed the study (mean 6.0, SD 8.7; *p* < 0.001).

**TABLE 1:** Baseline description of the FINGER population per randomization arm.

Demographics	*N*	Control^a^	Intervention^a^	*p* value
Age (years)	1174	69.0 (4.7)	69.5 (4.7)	0.09
Sex (women)	1174	47% (276)	44% (257)	0.20
Years of education	1174	10.1 (3.5)	10.0 (3.5)	0.67
**Cardiovascular risks and medical history**				
BMI	1165	28.1 (4.9)	28.3 (4.5)	0.71
Diabetes	1174	14% (79)	14% (82)	0.85
Cholesterol	1170	5.1 (1.0)	5.2 (1.0)	0.74
TBI	1174	12% (70)	15% (90)	0.10
Depressive symptoms	1174	22% (131)	20% (119)	0.34
**Lifestyle**				
Smoking *Never*	1174	50% (292)	49% (287)	0.46
*Ever*		42% (244)	41% (243)	
*Current*		8% (48)	10% (60)	
Alcohol consumption (moderate)	1174	72% (419)	72% (424)	0.96
Fish consumption (portions/week)	1174	1.7 (1.2)	1.7 (1.0)	0.90
Social engagement *Lowest*	1174	25% (144)	27% (162)	0.14
*Low to medium*		24% (140)	23% (137)	
*Medium to high*		25% (148)	28% (166)	
*Highest*		26% (152)	21% (125)	
Cognitive activity *Lowest*	1174	34% (200)	36% (215)	0.23
*Middle*		31% (181)	32% (191)	
*Highest*		35% (2013	31% (184)	
Physical activity *Lowest*	1174	28% (166)	30% (178)	0.73
*Middle*		41% (242)	36% (215)	
*Highest*		30% (176)	33% (197)	
**Cognition**				
Total composite score	1174	0.03 (0.59)	−0.03 (0.56)	0.10
Memory	1174	0.03 (0.66)	−0.03 (0.69)	0.12
Executive functioning	1174	0.02 (0.70)	−0.03 (0.66)	0.25
Processing speed	1174	0.04 (0.84)	−0.03 (0.79)	0.17
**ANU‐ADRI score**				
Full score	1174	5.9 (8.7)	6.9 (8.7)	0.06
Without age and sex	1174	−1.4 (5.2)	−1.0 (5.4)	0.21
ANU‐ADRI risk factors sub‐score at baseline	1174	14.8 (8.2)	15.8 (8.3)	0.04
ANU‐ADRI protective factors sub‐score at baseline	1174	−8.9 (2.6)	−8.9 (2.7)	0.87

*Note*: *t* test for continuous, *χ* ^2^ for dichotomous and rank sum for ordinal variables were used.

Abbreviations: ANU‐ADRI, Australian National University Alzheimer's Disease Risk Index; BMI, body mass index; TBI, traumatic brain injury.

^a^ Values expressed as either mean (standard deviation) or percentage (number of cases).

### Association between ANU‐ADRI and cognition in the FINGER intervention

The associations between (a) baseline ANU‐ADRI risk score and baseline cognition; (b) baseline ANU‐ADRI and changes in cognition; and (c) changes in ANU‐ADRI and changes in cognition were investigated throughout the 2‐year intervention considering both the total NTB composite score and the individual cognitive domains.

For all cognitive outcomes, significant associations were found cross‐sectionally at baseline (e.g., estimate for the cognitive total composite score [95% confidence interval, CI] was −0.028 [−0.032 to −0.025], Table 2a), between baseline ANU‐ADRI and per‐year changes in cognition (e.g., estimate for per‐year changes in the cognitive total composite score [95% CI] was −0.004 [−0.006 to −0.003], Table 2b) as well as between dichotomous (decreased vs. no change/increased) 2‐year changes in ANU‐ADRI risk score and per‐year changes in cognition (e.g., estimate for per‐year changes in the cognitive total composite score [95% CI] was −0.068 [−0.026 to −0.108], Table 2a). The direction of the association estimates consistently indicated that a higher/increased ANU‐ADRI risk score significantly correlated with worse cognition (Table 2).

**TABLE 2:** Association between ANU‐ADRI and cognition in the FINGER trial.

Domain	*N*	BL ANU‐ADRI and cognition^a^		BL ANU‐ADRI and per‐year changes in cognition^b^		2‐year change in ANU‐ADRI and per‐year changes in cognition^c^	
		Estimate (CI)	*p* value	Estimate (CI)	*p* value	Estimate (CI)	*p* value
Total composite	975	−0.028 (−0.032 to −0.025)	<0.001	−0.004 (−0.006 to −0.003)	<0.001	−0.068 (−0.026 to −0.108)	0.001
Memory	975	−0.021 (−0.026 to −0.017)	<0.001	−0.006 (−0.008 to −0.004)	<0.001	−0.100 (−0.030 to −0.168)	0.005
Executive function	975	−0.030 (−0.034 to −0.025)	<0.001	−0.003 (−0.005 to −0.002)	<0.001	−0.058 (−0.005 to −0.110)	0.032
Processing speed	975	−0.038 (−0.043 to −0.033)	<0.001	−0.004 (−0.006 to −0.003)	<0.001	−0.082 (−0.024 to −0.0138)	0.005

*Note*: Estimates and 95% CI measures from a mixed model with maximum likelihood estimation, with cognition as outcome as a function of ANU‐ADRI, adjusted for site. For each cognitive outcome, the results are reported from the same model and include participants for whom the ANU‐ADRI score was available at both baseline and 2 years (*n* = 975). The model included randomization group, time in years, baseline ANU‐ADRI risk score and the interactions time × randomization group, time × baseline ANU‐ADRI risk score and time × dichotomous changes in ANU‐ADRI risk scores, adjusted for study site.

Abbreviations: ANU‐ADRI, Australian National University Alzheimer's Disease Risk Index; BL, baseline; CI, confidence interval.

^a^ Association between baseline ANU‐ADRI risk score and baseline cognition; a negative estimate indicates that a higher ANU‐ADRI risk score is associated with lower cognition.

^b^ Association between baseline ANU‐ADRI and per‐year changes in cognition; a negative estimate indicates that a higher ANU‐ADRI risk score at baseline is associated with decreasing cognition over time.

^c^ Association between 2‐year dichotomous changes in ANU‐ADRI risk score (without age and sex) and change in cognition; a negative association indicates that decreasing ANU‐ADRI risk score is associated with better cognition over time.

### Intervention effects on changes in ANU‐ADRI

**FIGURE 1:**
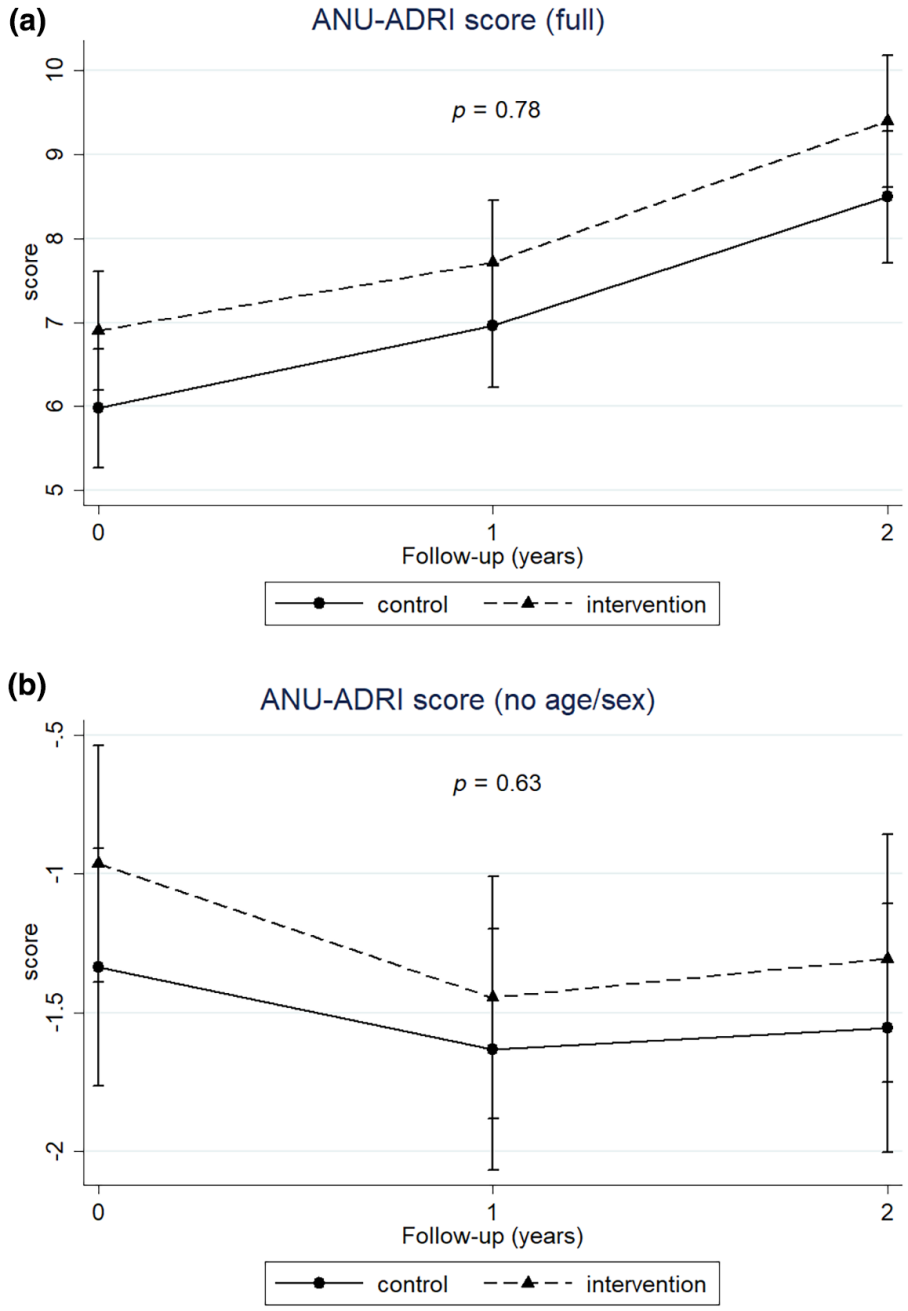
Changes in ANU‐ADRI as full risk score (a) and without age and sex (b) per randomization arm during the 2‐year intervention. Mixed model repeated measures with maximum likelihood estimation as a function of randomization, time and their interaction, adjusted per site. Time as categorical variable (overall *p* value reported). A lower ANU‐ADRI score value indicates a better outcome.

**FIGURE 2:**
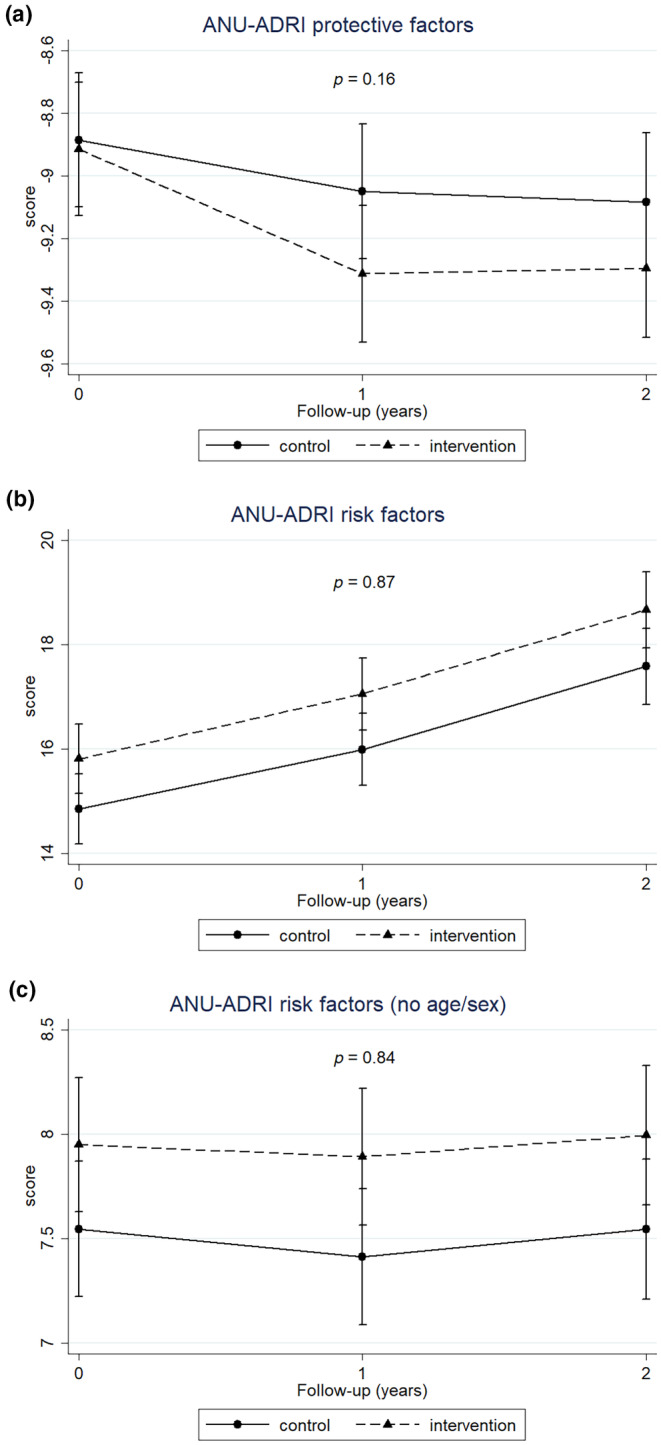
Changes in ANU‐ADRI protective factors sub‐score (a) and risk sub‐scores ((b) full; (c) without age and sex) and during the 2‐year intervention. Mixed model repeated measures with maximum likelihood estimation as a function of randomization, time and their interaction, adjusted per site. Time as categorical variable (*p* value reported for continuous time). For both risk and protective factors, a reduction in the sub‐scores indicates a better outcome.

Observed changes in ANU‐ADRI risk score across the 2‐year intervention are shown in Table S3. The full score (Figure 1a, Tables S3 and S4) increased for both groups with no significant difference. When age and sex, which are non‐modifiable factors and have a substantial weight in the overall score composition, were not included, a score decrease was reported during the first‐year intervention for both groups, but without significant beneficial effect of the intervention compared to the control (Figure 1b, Tables S4 and S5). Similar results were obtained when estimating intervention effects on risk or protective sub‐scores (Figure 2, Tables S6 and S7) and, although a beneficial trend was observed on the protective sub‐score for the intervention (Figure 2c), no significant differences in the level of *p* < 0.05 were reported between intervention and control.

The intervention effects were also estimated on all individual modifiable components of the ANU‐ADRI risk score, using the same statistical model. A significant intervention effect was reported for physical activity (−0.22 points; *p* = 0.003) but not for other individual components.

### Modifying effect of ANU‐ADRI in the intervention effect on changes in cognition

The interaction between time, randomization group and baseline ANU‐ADRI, included as a factor in the mixed model assessing intervention effects on the trial primary outcome, was not significant (*p* = 0.34), indicating that the baseline ANU‐ADRI did not significantly affect the response to the intervention on changes in cognition.

## DISCUSSION

In this post hoc analysis of the FINGER trial, in which a multidomain lifestyle intervention was shown to reduce the risk of cognitive decline, significant and consistent associations were reported between ANU‐ADRI and the cognitive primary and secondary outcomes, both at baseline and over the 2‐year study period. The FINGER intervention did not significantly modify the ANU‐ADRI risk score, nor its risk and protective sub‐scores, and baseline ANU‐ADRI did not modify the beneficial intervention effect on cognition.

Taken together, these findings confirmed the reliable risk estimation capacity of ANU‐ADRI, in a cohort of older adults at increased risk of dementia from Finland. This supports its possible use as a stratification tool for selecting participants in targeted multidomain RCTs and monitoring for wider prevention programmes at individual and population level. However, the findings related to the effect of the FINGER intervention on ANU‐ADRI suggested that, in the FINGER study‐specific context, ANU‐ADRI may not be a sensitive surrogate outcome.

Multidomain lifestyle interventions (like FINGER) are based on the hypotheses that individual changes in multiple risk factors, albeit small, can lead to concrete health benefits when combined together, even when different factors are modified in different people [24]. Risk scores, which are not the mere sum of different risk/protective factors but reflect risk profiles in weighted combination of specific factors for each individual [9], could be particularly suitable to capture such benefits.

Results from previous analysis on the effect of the FINGER intervention on changes in CAIDE [11] and Lifestyle for Brain Health (LIBRA) [10] dementia risk scores support this suggestion, together with the notion that a reduction in overall dementia risk may be obtained by multidomain preventive approaches. In FINGER, the CAIDE risk score also correlated to neuroimaging markers of cerebrovascular change and neurodegeneration [25, 26]. However, different risk scores, combining different factors in different mathematical models, may reflect different aspects of an individual's risk profile. For example, CAIDE and LIBRA risk scores are heavily based on vascular and lifestyle risk factors which are also very relevant components for the FINGER intervention; others may also focus on additional factors that are related to, for example, medical history or socio‐economic status [9]. The CAIDE risk score was also used in the selection of participants for FINGER and was developed on a similar population from Finland. The LIBRA score was developed from a cohort from the Netherlands, that is, the Maastricht Ageing Study. It is possible that greater similarity between development cohorts and intervention cohorts increases the sensitivity of measures.

The lack of intervention effect on changes in the ANU‐ADRI risk score suggests that its algorithm and the specific components of the FINGER intervention may not be sufficiently aligned for the actual intervention‐led changes in risk profile, which are linked to benefits in cognition, to be suitably captured by this risk score. First, factors such as BMI and cholesterol are not included in the calculation of the ANU‐ADRI risk score due to the population age range. The FINGER intervention was designed to impact on these factors and the study found a significant intervention effect on BMI [23].

Furthermore, changes in the cognitive training and the social components of the FINGER intervention could not be specifically measured with the tools available for data collection and therefore they did not translate into specific changes in the related components of the ANU‐ADRI algorithm, especially in the case of social engagement. Additionally, the ANU‐ADRI risk score was designed as a comprehensive score comprising a wide range of factors, and some of them (traumatic brain injury, depression, pesticide exposure) were not specifically targeted by the FINGER intervention, which was designed to largely focus on lifestyle, vascular and metabolic risk factors.

Nonetheless, the consistent association between the ANU‐ADRI risk score and cognitive performance during the FINGER trial showed that this risk score reflects cognition trajectories over time. Additionally, the results reported on the risk/protective factors separately, although not significant, suggest that the protective sub‐score, including more lifestyle/vascular factors (e.g., physical activity, fish and moderate alcohol intake), might perform better in capturing intervention changes. Many methodological aspects can impact on the choice of the most appropriate risk score for a certain context, including specifics of the intervention design, the addressed modifiable factors, and characteristics of the target population [8]. In particular, non‐modifiable risk factors, such as age and genetics, also play a crucial role in defining individual risk profiles [27, 28] and can significantly affect the performance of a risk score in a certain population. For example, the results obtained on the intervention effect of the ANU‐ADRI protective sub‐risk score, without age and sex, suggest that, in this population of older adults, the substantial weight of age in the overall score may have partially ‘masked’ the intervention‐led beneficial changes on the changes in risk profile over time.

The main strength of this study is the assessment, for the first time, of ANU‐ADRI, one of the most comprehensive tools for AD risk estimation, in FINGER, the first larger RCT to show a beneficial effect on cognition and dementia risk reduction of a multidomain intervention targeting lifestyle, vascular and metabolic risk factors in a population of older adults with risk factors for dementia. The fact that the FINGER intervention was indeed able to reduce cognitive decline over the 2‐year period allowed the performance of the ANU‐ADRI risk score to be assessed more accurately, both as a risk estimation tool and as a surrogate outcome. Also, ANU‐ADRI includes a wide range of factors which allowed its association with cognitive performance to be consistently captured.

The study was mostly limited by the need to adapt the ANU‐ADRI algorithm to the available dataset as the use of the ANU‐ADRI was not part of the original study design, which may have affected its performance and prevented its full potential from being assessed. This translated in the exclusion of important risk factors for the FINGER intervention (e.g., BMI), and the use of different tools for the measurement of a certain component (e.g., the Zung scale for depression, instead of the Center for Epidemiological Studies Depression scale) or alternative questions for self‐reported factors (e.g., social engagement questions, which measured social activities but not less formal social involvement). Additionally, since ANU‐ADRI includes a wide range of risk/protective factors (15 in the original algorithm) and all factors included were needed for the calculation of the risk score, this resulted in 85 participants not being included in the present analysis due to missing data. The lack of predefined scales for social and cognitive activities meant that these components had also to be scaled to the FINGER population rather than in absolute terms, and pesticide exposure could not be included due to data unavailability. This may have hampered the performance of the risk score in capturing intervention‐led changes and affected the overall results. It is also possible that the effect size of the FINGER intervention was not detectable with the ANU‐ADRI version used in this study. Due to these limitations, the potential use of ANU‐ADRI as a surrogate outcome cannot be fully generalized to other multidomain lifestyle interventions based on these results alone. This is supported by the results from the four small trials published to date that included the ANU‐ADRI instrument in their design, used the ANU‐ADRI questionnaire as an outcome measure, and found a significant effect of the intervention [16, 17, 18, 19].

The fact that the modified baseline ANU‐ADRI score and the risk factors score were higher for the intervention group in at least a borderline significant manner is also a possible limitation, as it can skew the results to show less of an intervention effect for ANU‐ADRI. Additionally, since FINGER participants were already selected at baseline to be at increased risk for dementia compared to the general population, this could have reduced the potential for variability of the ANU‐ADRI across participants.

In conclusion, the ANU‐ADRI score was confirmed as an effective tool for the risk prediction of cognitive decline. When appropriately chosen, risk scores may be instrumental in the design and implementation of novel successful trials aimed at dementia prevention or dementia risk reduction. In particular, in order to capture changes in specific risk profiles, risk scores used as surrogate outcomes to test the prevention potential of complex multidomain lifestyle interventions must be identified with careful consideration for their mathematical model, the target population and the intervention design.

## AUTHOR CONTRIBUTIONS

**Mariagnese Barbera:** Conceptualization; writing – original draft; writing – review and editing. **Anette Hall:** Conceptualization; formal analysis; writing – original draft; writing – review and editing. **Jenni Lehtisalo:** Conceptualization; writing – review and editing. **Riitta Antikainen:** Funding acquisition; writing – review and editing. **Hamidul Huque:** Conceptualization; writing – review and editing. **Tiina Laatikainen:** Funding acquisition; writing – review and editing. **Tiia Ngandu:** Funding acquisition; writing – review and editing. **Hilkka Soininen:** Funding acquisition; writing – review and editing. **Ruth Stephen:** Conceptualization; writing – review and editing. **Timo Strandberg:** Funding acquisition; writing – review and editing. **Miia Kivipelto:** Funding acquisition; writing – review and editing. **Kaarin J. Anstey:** Conceptualization; funding acquisition; writing – review and editing. **Alina Solomon:** Conceptualization; funding acquisition; formal analysis; writing – review and editing.

## FUNDING INFORMATION

This work was supported by grants from Alzheimerfonden (Sweden); Region Stockholm (ALF grant); European Research Council (ERC, 804371); EU Joint Programme—Neurodegenerative Disease Research (JPND) EURO‐FINGERS and Multi‐MeMo grants; NordForsk NJ‐FINGERS grant; Swedish Research Council; Ella and Georg Ehrnrooth Foundation; Finnish Cultural Foundation; Center for Innovative Medicine (CIMED) at Region Stockholm (Sweden); Stiftelsen Stockholms sjukhem (Sweden); Swedish Research Council for Health, Working Life and Welfare (FORTE); Hjärnfonden (Sweden); Research Council of Finland; Juho Vainio Foundation (Finland); Finnish Cultural Foundation (Finland); Ministry of Education and Culture (Finland); Yrjö Jahnsson Foundation (Finland); Alzheimer's Research and Prevention Foundation (US); Kela (Finland); Finnish Foundation for Cardiovascular Research (Finland); Sigrid Jusélius Foundation (Finland); EVO grants of Oulu; KJA is funded by ARC Laureate Fellowship FL190100011.

## CONFLICT OF INTEREST STATEMENT

HS reports personal payment for being a steering group member of the EVOKE and EVOKE+ trials. The other authors have stated explicitly that there are no conflicts of interest in connection with this article.

## Supporting information


**Appendix S1.** Supporting Information.

## Data Availability

Data used in this study are not publicly available due to ethical and legal reasons, but the data are available upon request. Those fulfilling the requirements for viewing confidential data as required by the Finnish legislation and the Finnish Institute for Health and Welfare are able to access the data after completion of a material transfer agreement. Requests may be directed to kirjaamo@thl.fi.
