# Supplementary material for: The Australian National University Alzheimer's Disease Risk Index (ANU‐ADRI) score as a predictor for cognitive decline and potential surrogate outcome in the FINGER lifestyle randomized controlled trial
Source: Eur J Neurol. 2024 Feb 7;31(5):e16238. doi: 10.1111/ene.16238 (PMC11235774; doi:10.1111/ene.16238)
Supplement: Supplementary file 1 — Appendix S1. Supporting Information. [file ENE-31-e16238-s001.docx]

**Hall et al. “The ANU-ADRI risk score as a predictor for cognitive decline and potential surrogate outcome in the FINGER lifestyle randomized controlled trial”**

**SUPPLEMENTARY MATERIAL**

**Supplementary Methods. Assessment of Social and Cognitive activities in the FINGER trial.**

Social Engagement and Cognitive Activities were self-reported by participants through a questionnaire that enquired about the following activities:

- ***Social activities***: playing games in a group; attending clubs or other hobby-groups; attending courses/studying in a group; baby-sitting; doing volunteering activities.
- ***Cognitive activities***: reading; doing crossword puzzles; writing; playing games (in a group); listening/playing music; attending courses/studying (in a group); doing arts and craft; gardening.

Participants were asked how often they engaged in each of these activities in their normal life and the possible options for answer were:

1. Daily
2. 4-6 times a week
3. 2-3 times a week
4. Once a week
5. 2-3 times a month
6. A few times a year or less
7. Never

***Calculation of the Social Engagement and Cognitive Activities components of ANU-ADRI***. For all activities, each option was assigned a sequential numerical score ranging 1-7, as shown above, and the numerical scores of activities included in the relevant ANU-ADRI components were summed to obtain an “overall score” for Social Engagement and one for Cognitive Activities. Social Engagement and Cognitive Activities ANU-ADRI components were then calculated based on the quartile (Social Engagement) or tertile (Cognitive Activities) distribution of the respective baseline “overall scores” (Supplementary Table 1). When missing data were reported for one of the activities included one of the two ANU-ADRI components, imputations were applied to calculate the “overall score” for that specific component, so that the numerical score of the missing activity was calculated as the mean of that specific activity across all participants. The “overall score” was not calculated, and the related ANU-ADRI component was considered missing, if missing data were reported for more than one activity included in either specific component (Social Engagement or Cognitive Activities).

**Supplementary Table 1. Algorithm for the calculation of the ANU-ADRI within the FINGER cohort.**

| **Risk/protective factor** | **FINGER assessment method** | **Risk categories** | **Points** |
| --- | --- | --- | --- |
| *Age and sex* | Age: calculated from DOB Sex: self-reported | < 65 years | 0 |
|  |  | 65-70 years | 1 (m) ; 5 (w) |
|  |  | 71-75 years | 12 (m); 14 (w) |
|  |  | 76-80 years | 18 (m); 21 (w) |
| *Education* | Self-reported years of formal education | >11 years | 0 |
|  |  | 8-11 years | 3 |
|  |  | <8 years | 6 |
| *Diabetes* | Self-reported diagnosis or drug prescription | No | 0 |
|  |  | Diabetes diagnosis/treatment diabetes | 3 |
| *Depression* | Zung depression scale* | Zung score < 40 | 0 |
|  |  | Zung score ≥ 40 | 2 |
| *TBI* | Self-reported history of TBI | No | 0 |
|  |  | Yes | 4 |
| *Smoking* | Self-reported smoking status | Never smoker | 0 |
|  |  | Past smoker | 1 |
|  |  | Current smoker | 4 |
| *Alcohol* | Self-reported frequency of alcohol consumption | No consumption/above the recommended values^a^ | 0 |
|  |  | Within the recommended values^a^ | -3 |
| *Social Engagement^b^* | Self-reported frequency of engagement in social activities (games; clubs; studying; baby-sitting; volunteering) | 1st quartile | 0 |
|  |  | 2nd quartile | 1 |
|  |  | 3rd quartile | 4 |
|  |  | 4th quartile | 6 |
| *Physical activity* | Self-reported weekly frequency of at least moderate intensity exercise sessions | 1 or less | 0 |
|  |  | 2-3 | -2 |
|  |  | 4 or more | -3 |
| *Cognitive activity^b^* | Self-reported frequency of cognitive activities (reading; crossword puzzles; writing; games; music; studying; arts and craft; gardening) | 3rd tertile | 0 |
|  |  | 2nd tertile | -6 |
|  |  | 1st tertile | -7 |
| *Fish consumption* | Self-reported weekly consumption (portions of fish as main course) | < 0.25 | 0 |
|  |  | 0.26 - 2.00 | -3 |
|  |  | 2.01 - 4.00 | -4 |
|  |  | > 4.00 | -5 |

Note.

1. Based on the Finnish maximum recommended level of alcohol consumption for older adults (7 units/week)
2. Full details of the methods to evaluate social and cognitive activities are provided in Supplementary Methods.

DOB: date of birth; m: men; TBI: traumatic brain injury; w: women.

**Supplementary Table 2. Tests and scores included in the Neuropsychological Test Battery conducted within FINGER.**

| **Cognitive Test** | **Item/Score Included** | **Cognitive Domain** |
| --- | --- | --- |
| WMSR - Logical Memory | Immediate recall | Memory |
| WMSR - Logical Memory | Delayed recall |  |
| WMSR - Visual Paired Associates | Immediate recall |  |
| WMSR - Visual Paired Associates | Delayed recall |  |
| CERAD Word List | Learning score |  |
| CERAD Word List | Recall score |  |
| CERAD Category Fluency | Number of correct animals listed | Executive Function |
| Digit Span | Total score |  |
| Trail Making Tests | Shifting score B-A |  |
| Concept Shifting Test | Condition B |  |
| Stroop | Interference score condition 3-2 |  |
| Concept Shifting Test | Condition A | Processing speed |
| Stroop | Condition 2 |  |
| Letter-Digit Substitution Test | Total score |  |

CERAD: Consortium to Establish a Registry for Alzheimer’s Disease; WMSR: Wechsler Memory Scale Revised.

**Supplementary Table 3. Observed ANU-ADRI changes across the FINGER trial time points per randomization arm (T-test).**

|  | **N** | **Control^a^** | **Intervention^a^** | **P-value** |
| --- | --- | --- | --- | --- |
| **Full score**  Baseline | 1174 | 5.9 (8.7) | 6.9 (8.7) | 0.06 |
| 12-Month | 1021 | 6.8 (9.1) | 7.4 (9.3) | 0.24 |
| 24-Month | 975 | 8.0 (9.4) | 8.8 (9.5) | 0.19 |
| Baseline to 12-Month change | 1021 | 1.0 (4.4) | 0.8 (4.4) | 0.40 |
| Baseline to 24-Month change | 975 | 2.5 (5.0) | 2.4 (5.5) | 0.94 |
| **Without age and sex**  Baseline | 1174 | -1.4 (5.2) | -1.0 (5.4) | 0.21 |
| 12-Month | 1021 | -1.7 (5.4) | -1.7 (5.3) | 0.85 |
| 24-Month | 975 | -1.7 (5.4) | -1.7 (5.3) | 0.90 |
| Baseline to 12-Month change | 1021 | -0.3 (3.2) | -0.5 (3.3) | 0.34 |
| Baseline to 24-Month change | 975 | -0.2 (3.2) | -0.4 (3.5) | 0.50 |

Note. a. Values expressed as mean (standard deviation).

**Supplementary Table 4. Estimated intervention changes and effect on the ANU-ADRI score with time as a categorical variable.**

| **Changes in ANU-ADRI score** | **Score version** | **Estimated Mean Changes (CI)** | | **Difference between intervention and control groups** | |
| --- | --- | --- | --- | --- | --- |
|  |  | **Control** | **Intervention** | **Estimated Effect (95% CI)** | **P-value** |
| **From BL to 12M** | Full | 0.99 (0.61 to 1.36) | 0.81 (0.43 to 1.20) | -0.17 (-0.71 to 0.36) | 0.53 |
| **From BL to 24M** | Full | 2.51 (2.06 to 2.97) | 2.49 (2.04 to 2.95) | -0.02 (-0.67 to 0.62) | 0.95 |
| **From BL to 12M** | No age and sex | -0.30 (-0.56 to -0.03) | -0.48 (-0.75 to -0.21) | -0.19 (-0.57 to 0.19) | 0.34 |
| **From BL to 24M** | No age and sex | -0.22 (-0.51 to 0.07) | -0.34 (-0.64 to -0.05) | -0.12 (-0.54 to 0.29) | 0.56 |

Note. Mixed model repeated measures with maximum likelihood estimation as a function of randomization, time, and “randomization × time” interaction, adjusted per site. Time as categorical variable.

BL: baseline; M: months.

**Supplementary Table 5. Estimated intervention changes and effect on the ANU-ADRI score with time as a continuous variable.**

|  | **Estimated Mean Changes (CI) per year** | | **Difference between intervention and control groups per year** | |
| --- | --- | --- | --- | --- |
|  | **Control** | **Intervention** | **Estimated Effect (95% CI)** | **P-value** |
| **ANU-ADRI Full score** | 1.25 (1.02 to 1.47) | 1.23 (1.00 to 1.46) | -0.02 (-0.34 to 0.31) | 0.92 |
| **ANU-ADRI Without age and sex** | -0.12 (-0.26 to 0.03) | -0.18 (-0.33 to 0.04) | -0.07 (-0.27 to 0.14) | 0.53 |

Note. Mixed model repeated measures with maximum likelihood estimation as a function of randomization, time, and “randomization × time” interaction, adjusted per site. Time as continuous variable.

**Supplementary Table 6. Estimated intervention changes and effect on the ANU-ADRI risk- or protective-factors sub-scores with time as a categorical variable.**

| **Changes in ANU-ADRI sub-score** | **Sub-core version** | **Estimated Mean Changes (CI)** | | **Difference between intervention and control groups per year** | |
| --- | --- | --- | --- | --- | --- |
|  |  | **Control** | **Intervention** | **Estimated Effect (95% CI)** | **P-value** |
| **From BL to 12M** | Risk sub-score full | 1.14 (0.82 to 1.45) | 1.24 (0.93 to 1.56) | 0.11 (-0.34 to 0.55) | 0.64 |
| **From BL to 24M** | Risk sub-score full | 2.73 (2.34 to 3.12) | 2.86 (2.46 to 3.25) | 0.13 (-0.43 to 0.68) | 0.65 |
| **From BL to 12M** | Risk sub-score no age and sex | -0.14 (-0.31 to 0.04) | -0.06 (-0.23 to 0.12) | 0.08 (-0.17 to 0.33) | 0.60 |
| **From BL to 24M** | Risk sub-score no age and sex | 0.00 (-0.20 to 0.19) | 0.04 (-0.15 to 0.24) | 0.05 (-0.23 to 0.32) | 0.32 |
| **From BL to 12M** | Protective sub-score full | -0.16 (-0.34 to -0.01) | -0.40 (-0.58 to 0.22) | -0.23 (-0.48 to 0.02) | 0.07 |
| **From BL to 24M** | Protective sub-score full | -0.20 (-0.39 to -0.01) | -0.38 (-0.57 to -0.20) | -0.18 (-0.45 to 0.08) | 0.17 |

Note. Mixed model repeated measures with maximum likelihood estimation as a function of randomization, time, and “randomization × time” interaction, adjusted per site. Time as categorical variable.

BL: baseline; M: months.

**Supplementary Table 7. Estimated intervention changes and effect on the ANU-ADRI risk- and protective- factors sub-scores with time as a continuous variable.**

|  | **Estimated Mean Changes (CI)** | | **Difference between intervention and control groups per year** | |
| --- | --- | --- | --- | --- |
|  | **Control** | **Intervention** | **Estimated Effect (95% CI)** | **P-value** |
| **Protective sub-score** | -0.10 (-0.19 to -0.01) | -0.20 (-0.39 to -0.10) | -0.09 (-0.23 to 0.04) | 0.16 |
| **Risk sub-score** | 1.36 (1.16 to 1.55) | 1.42 (1.23 to 1.62) | 0.07 (-0.21 to 0.34) | 0.64 |
| **Risk sub-score (no age/sex)** | -0.00 (-0.10 to 0.09) | 0.02 (-0.08 to 0.12) | 0.02 (-0.11 to 0.16) | 0.73 |

Note. Mixed model repeated measures with maximum likelihood estimation as a function of randomization, time, and “randomization X time” interaction, adjusted per site. Time as continuous variable.

stylefix
